# Association of serum thymosin β4 with malnutrition-inflammation-atherosclerosis syndrome in peritoneal dialysis patients: a cross-sectional study

**DOI:** 10.1080/0886022X.2023.2202761

**Published:** 2023-05-03

**Authors:** Jiakun Tian, Rong Zhang, Nan Zhu, Lijie Gu, Yunshan Guo, Weijie Yuan

**Affiliations:** a Department of Nephrology, Shanghai General Hospital of Nanjing Medical University, Shanghai, China; b Department of Nephrology, Shanghai General Hospital, Shanghai Jiao Tong University School of Medicine, Shanghai, China

**Keywords:** Thymosin β4, peritoneal dialysis, malnutrition-inflammation-atherosclerosis syndrome

## Abstract

**Background:**

Malnutrition-inflammation-atherosclerosis (MIA) syndrome may worsen the prognosis of peritoneal dialysis (PD) patients. Serum thymosin β4 (sTβ4) protects against inflammation, fibrosis and cardiac dysfunction.

**Objectives:**

The present study aimed to characterize the association between sTβ4 and MIA syndrome as well as to investigate the potential of regulating sTβ4 to improve the prognosis of PD patients.

**Methods:**

We performed a cross-sectional, single-center pilot study involving 76 PD patients. Demographic characteristics, clinical characteristics, nutritional profiles, inflammatory mediators, atherosclerosis-related factors and sTβ4 levels were collected and subjected to association analysis for sTβ4 and MIA syndrome.

**Results:**

sTβ4 levels did not significantly vary with sex or primary disease in PD patients. Ages and PD features did not vary between patients with different levels of sTβ4. PD patients with higher levels of sTβ4 had significantly higher levels of nutritional indicators, including subjective global nutritional assessment (SGA) (*p* < 0.001) and serum albumin (ALB) (*p* < 0.001) but lower levels of inflammatory and atherosclerotic indicators, including serum C reaction protein (CRP) (*p* = 0.009), the right common carotid artery (RCCA) intimal thickness (*p* < 0.001) and the left common carotid artery (LCCA) intimal thickness (*p* = 0.02). Correlation analysis showed that sTβ4 was positively associated with SGA (*p* < 0.001) and serum ALB (*p* < 0.001) but negatively associated with CRP (*p* = 0.020), RCCA intimal thickness (*p* < 0.001) and LCCA intimal thickness (*p* = 0.033). In multiple adjusted models, the prevalence of MIA syndrome was significantly decreased in PD patients with increased levels of sTβ4 when patients without MIA syndrome were compared to those with all indicators of MIA syndrome (OR = 0.996, 95% CI 0.993–0.999, *p* = 0.003) or those with at least one indicator of MIA syndrome (OR = 0.997, 95% CI 0.995–0.998, *p* < 0.001).

**Conclusions:**

The sTβ4 level decreases in PD patients with MIA syndrome. The prevalence of MIA syndrome decreases significantly as the level of sTβ4 increases in PD patients.

## Introduction

As a main modality of kidney replacement therapy, peritoneal dialysis (PD) has benefited approximately 10–15% of end-stage kidney disease (ESKD) patients worldwide [1]. However, atherosclerosis (AS) remains a leading cause of deteriorated prognosis and all-cause mortality in PD-treated ESKD patients. AS and AS-related heart diseases, including cardiovascular disease (CVD) and heart failure, account for nearly 50% of deaths in these patients [2,3]. Endogenous and exogenous assaults, such as uremic toxins, blood lipid metabolism disorders, implanted catheters and glucose-based solutions, may increase the risks of AS, inflammation and malnutrition in PD patients [4]. These three abnormalities interact with each other in ESKD patients, inducing malnutrition-inflammation-atherosclerosis (MIA) syndrome, which further impairs the quality of life and prognosis of PD patients [5,6].

As a natural peptide and a conserved plasmosin with a medium molecular weight of 4963.55 D, thymosin β4 (Tβ4) contains multiple essential amino acids and is edited by the X chromosome TMSB4X [7]. Synthesized by macrophages and endothelium in various organs, such as the thymus, kidneys, liver, lungs and brain, Tβ4, as a highly conserved G-actin sequestering protein, is closely implicated in anti-inflammation, anti-fibrosis, cardioprotection and heart repair. Exogenous Tβ4 exerts inhibitory effects in diverse pathologies, including myocardial infarction, inflammatory lung disease and kidney fibrosis. Animal and clinical trials have assessed the protective effects of Tβ4 treatment on pulmonary, renal and cardiac functions. Tβ4 attenuates oxidative injury, inflammation and fibrosis; thus, Tβ4 may be modulated to counter pulmonary fibrosis. Exogenous Tβ4 has demonstrated therapeutic benefits in animal models of interstitial fibrosis and diabetic nephropathy. Tβ4 facilitates cardiac repair after infarction by promoting cell migration and myocyte survival. Moreover, Tβ4 has been shown to modulate several cellular functions, including cell motility, cell differentiation, cell survival, angiogenesis, inflammation and fibrosis [8–12]. Recently, Tβ4 has been detected in the effluent dialysate of hemodialysis and demonstrated to be associated with systemic inflammation and kidney fibrosis [13]. Thus, we hypothesize that sTβ4 is related to MIA syndrome in PD patients. Therefore, we performed a cross-sectional study to analyze the following components: 1) the sTβ4 profile in PD patients; 2) the association between sTβ4 and MIA syndrome; and 3) the protective role of Tβ4 in PD patients.

## Materials and methods

### Study design and subjects

This cross-sectional, single-center study was supported by the National Natural Science Foundation of China (Grant No: 82170729). The study involved 76 PD patients who had been treated with continuous ambulatory peritoneal dialysis (CAPD) for ≥3 months and regularly followed up at the Lianyungang Clinical College of Nanjing Medical University/The First People’s Hospital of Lianyungang. All patients received routine examinations of standardized peritoneal equilibration test (PET), total weekly Kt/V (Kt/V t), peritoneal weekly Kt/V (Kt/V p), residual kidney weekly Kt/V (Kt/V r), total weekly creatinine clearance rate (Ccr t; normalized to 1.73 m^2^ of the body surface area), peritoneal Ccr (Ccr p) and kidney Ccr (Ccr r). All patients were in stable condition, and they were free of peritonitis and clinical and/or laboratory signs of other infections during the one month prior to enrollment. Those with a history of malignant diseases, malignant tumors, illness or hospitalization in the previous month were excluded. All included patients received a commercially available, glucose-based PD solution (Baxter Inc.) at various concentrations (1.5% and 2.5%). No patients received icodextrin-based PD solution because it was unavailable during the study period.

### Demographic and laboratory data

The demographic and clinical data included sex, primary disease, smoking history (SH), age, median time on PD, history of CVD, systolic blood pressure (SPB), diastolic blood pressure (DPB), subjective global nutritional assessment (SGA) and body mass index (BMI).

During regular clinical visits, morning whole-blood samples were collected from PD patients receiving PET, Kt/V and Ccr after overnight fasting. Partial blood samples were immediately centrifuged at 3000 rpm for 10 min and then stored at −80 °C until the determination of serum carbohydrate antigen 125 (CA125) and sTβ4 concentrations. Serum concentrations of hemoglobin (HGB), reticulocyte (Ret), C reaction protein (CRP) and interleukin-6 (IL-6) were measured using an automated blood cell analyzer (Mindray BC-6800 Plus, Mindray, China). Serum concentrations of glucose (GLU), creatinine (Scr), urea nitrogen (BUN), albumin (ALB), prealbumin (PA), calcium (Ca), phosphorus (P), intact parathyroid hormone (iPTH), total cholesterol (CH), triglyceride (TG), high-density lipoprotein cholesterol (HDL-C), low-density lipoprotein cholesterol (LDL-C), free fatty acid (FF), uric acid (UA), actual bicarbonate (AB) and homocysteine (HCY) were measured using an automatic biochemical analyzer (Beckmann au5800, Beckmann, USA). Serum brain natriuretic peptide (BNP), folic acid and high sensitive troponin (hs-cTn) were measured using an automatic biochemical analyzer (Beckmann DXI800, Beckmann, USA). Serum pro-brain natriuretic peptide (pro-BNP) was measured using an automatic fluorescence immunoanalyzer (Vidas J, BioMerieux, France). Serum total iron binding capacity (TIBC), transferrin (TRF), iron saturation, serum ferritin (SF) and serum iron (SI) were measured using an automatic chemiluminescence analyzer (Cobas e602, Roche Group, Switzerland). Serum β2-macroglobulin (β2-MG) was measured using an automatic protein analyzer (Siemens BNII, Siemens, Germany). Serum superoxide dismutase (SOD), nitric oxide (NO) and neutrophil gelatinase-associated lipocalin (NGAL) were measured using an automatic biochemical analyzer (Beckmann au5832, Beckmann, USA). The intimal thickness of the right common carotid artery (RCCA) and the left common carotid artery (LCCA) was measured using a cardiac ultrasound machine (EPIQ 5, Philips, Netherland). PET, daily urine output (DUO), daily peritoneal ultrafiltration (DPU), Kt/V t, Kt/V p, Kt/V r, Ccr t, Ccr p and Ccr r were measured using routine methods. CA125 and sTβ4 levels were analyzed with commercial ELISA kits (BYabscience Biotechnology Co., Ltd., China). All biochemical and clinical parameters were routinely determined in the laboratory, Ultrasound Department and Peritoneal Dialysis Center in our hospital. A CRP value >10 mg/L indicated the presence of inflammation according to the evaluation criteria of our laboratory.

#### SGA and BIA measurements

The SGA in PD patients was graded according to weight variation, dietetic status, gastrointestinal symptoms, activity of daily living, nutritional requirements, subcutaneous fat and muscle wasting. The subcutaneous fat was measured according to the skinfold thickness of the triceps and subscapular thickness using skinfold calipers. Grades ≥25 were defined as well nourished; otherwise, they were defined as malnourished [14].

Multifrequency bioimpedance analysis (BIA) (Multiscan5000, Bodystat®, England) was also performed in 65 PD patients. Phase angle (PhA), overhydration index (OH) and extracellular water (ECW)/total body water (TBW) were used to assess the nutritional status and volume of overload. The Multiscan5000 was used to measure body resistance and reactance to electrical currents of 50 discrete frequencies, ranging from 5 to 1,000 kHz. Values of OH, PhA and ECW/TBW at a frequency of 50 kHz were considered. A value of ECW/TBW >0.39 was defined as overhydration according to the Multiscan5000 instructions.

### Statistical analysis

Normally distributed data are expressed as the mean ± SD, and skewed data are presented as the median and interquartile range (Q25–Q75). Categorical data are presented as a frequency (%). Demographic and laboratory measurements were compared with independent sample t tests, Mann–Whitney U tests, one-way ANOVA or chi-square tests. Pearson’s or Spearman’s correlation analysis (as appropriate) was used to evaluate relationships between sTβ4 and indicators of MIA syndrome. Univariate and multiple logistic regression analyses were performed to evaluate the influence of sTβ4 on MIA syndrome in PD patients. Calculations were performed using the Statistical Package for the Social Sciences (version 25.0; SPSS IBM., Chicago, IL, USA). A p value of less than 0.05 was considered to be significant.

## Results

### Demographic data

A total of 76 eligible PD patients were enrolled in the present study. All data were obtained immediately after enrollment. The level of sTβ4 was 970.80 (591.99, 1309.15) ng/mL. There were no significant differences between the following comparisons: males (*n* = 40) and females (*n* = 36) (1018.46 ± 408.57 vs. 956.32 [537.71, 1304.74] ng/mL) (*p* = 0.731); patients with diabetic kidney disease (*n* = 28) and nondiabetic kidney disease (*n* = 48) (1030.61 [714.83, 1494.57] vs. 962.94 [517.19, 1295.14] ng/mL) (*p* = 0.155); and patients (*n* = 36) with and without a history of smoking (*n* = 40) (986.83 ± 434.10 vs. 1114.70 [591.99, 1322.29] ng/mL) (*p* = 0.739).

### Relation of Demographic and laboratory characteristics to sTβ4

Patients were divided into the following two groups according to the median sTβ4 level: low sTβ4 group (sTβ4 < 970.80 ng/mL, *n* = 38) and high sTβ4 group (sTβ4 > 970.80 ng/mL, *n* = 38). Demographic and laboratory data were compared between the two groups.

The high sTβ4 group had significantly higher levels of SGA (*p* < 0.001) and ALB (*p* < 0.001) but lower levels of serum CRP (*p* = 0.009), serum IL-6 (*p* = 0.02), RCCA intimal thickness (*p* < 0.001) and LCCA intimal thickness (*p* = 0.02) (Table 1).

**Table 1.** Patient demographic and laboratory characteristics in relation to sTβ4.

Characteristics	Normal values	Low sTβ4 group (*n* = 38)	High sTβ4 group (*n* = 38)	t/Z/χ^2^	*p* Value
Age (years)	–	55.95 ± 13.80	57.53 ± 13.14	−0.51	0.61
PD duration (M)	–	14.25 (4.00, 60.25)	16.50 (14.00, 39.25)	−0.79	0.43
CVD (N/Y)	–	12/26	31/7	19.34	<0.001
SBP (mmHg)	–	144.47 ± 27.51	144.32 ± 24.08	0.03	0.98
DBP (mmHg)	–	80.50 (74.25, 92.75)	92.50 (70.00, 101.25)	−1.15	0.25
SGA	–	20.71 ± 3.99	28.50 ± 4.26	−8.22	<0.001
BMI	–	23.48 ± 4.01	27.47 ± 5.41	−3.65	0.001
HGB (g/L)	115–150 (F) /130–175 (M)	98.61 ± 20.83	98.08 ± 9.42	0.14	0.89
Ret (%)	0.5–1.5	1.71 ± 0.50	1.73 ± 0.64	−0.15	0.88
CRP (mg/L)	≤10	8.20 (3.85, 25.90)	3.25 (1.20, 9.68)	−2.74	0.009
IL-6 (pg/mL)	–6.61	6.54 (4.64, 15.98)	5.21 (3.58, 6.89)	−2.28	0.02
GLU (mmol/L)	3.9–6.1	4.64 (4.22, 5.15)	4.49 (4.07, 5.05)	−0.32	0.75
Scr (µmol/L)	57–111	871.44 ± 283.32	1062.77 ± 340.43	−2.66	0.01
BUN (mmol/L)	2.9–8.2	22.61 ± 7.78	22.51 ± 4.87	0.07	0.95
Albumin (g/L)	35–55	26.63 ± 3.70	29.94 ± 3.66	−3.93	<0.001
Prealbumin (mg/L)	200–400	245.49 ± 80.11	318.82 ± 96.07	−3.61	0.001
Calcium (mmol/L)	2.08–2.60	2.17 ± 0.19	2.19 ± 0.21	−0.35	0.73
Phosphorus (mmol/L)	0.90–1.34	1.73 ± 0.43	1.76 ± 0.49	−0.29	0.78
iPTH (pg/ml)	15–65	147.30 (69.73, 390.85)	166.00 (60.88, 432.03)	−0.06	0.95
Total cholesterol (mmol/L)	2.33–5.17	4.05 ± 1.58	3.62 ± 0.88	1.45	0.15
Triglyceride (mmol/L)	0.52–1.78	1.47 (1.06, 1.94)	1.21 (0.96, 1.64)	−0.86	0.39
HDL-C (mmol/L)	0.91–1.74	0.99 (0.87, 1.19)	0.98 (0.88, 1.21)	−0.27	0.79
LDL-C (mmol/L)	2.00–3.61	2.50 (1.77, 3.25)	2.03 (1.80, 2.45)	−2.15	0.03
Uric acid (μmol/L)	155–357 (F) /208–428 (M)	412.75 (343.13, 551.65)	402.90 (345.00, 467.60)	−0.62	0.54
Actual bicarbonate (mmol/L)	21–25	26.00 (23.75, 27.00)	27.00 (25.00, 28.00)	−1.61	0.11
TIBC (μmol/L)	50–77	39.91 (34.12, 43.47)	40.45 (37.47, 44.01)	−1.31	0.19
Transferrin (g/L)	2.00–3.60	2.05 ± 0.50	2.14 ± 0.34	−0.92	0.36
Iron saturation	0.33–0.35	0.25 (0.18, 0.37)	0.29 (0.19, 0.33)	−0.78	0.43
Serum ferritin (ng/ml)	13–150 (F) 30–400 (M)	276.40 (208.60, 397.78)	159.55 (25.60, 331.13)	−3.41	0.001
Serum iron (µmol/L)	10.6–36.7	10.30 (8.05, 13.43)	10.85 (6.58, 14.33)	−0.74	0.46
Folic acid (nmol/L)	7–45.1	9.80 (6.53, 23.60)	8.88 (6.25, 22.73)	−0.76	0.45
β2-MG (mg/L)	0.9–2.7	28.74 ± 10.87	28.59 ± 13.66	0.05	0.96
Free fatty acid (mmol/L)	0.1–0.6	0.27 (0.14, 0.45)	0.30 (0.23, 0.37)	−0.93	0.35
Homocysteine (μmol/L)	0–15	22.85 (19.18, 28.95)	21.71(16.93, 25.50)	−1.38	0.17
SOD (U/ml)	110–215	97.48 ± 6.15	107.03 ± 10.26	−4.92	<0.001
Nitric Oxide (μmL/L)	30–60	33.75 (25.93, 45.13)	40.85 (35.83, 47.05)	−3.17	0.002
NGAL (ng/ml)	37–180	719.80 (541.40, 983.33)	880.60 (692.35, 1103.30)	−1.65	0.10
BNP (pg/ml)	0–125	166.00 (54.75, 423.25)	202.50 (117.75, 338.25)	−0.67	0.51
Pro-BNP (pg/ml)	0–450	6728.00 (4586.00, 5346.00)	2941.50 (875.00, 5525.75)	−4.71	<0.001
hs-cTn (ng/ml)	0–17.5	0.16 ± 0.05	0.08 ± 0.04	7.00	<0.001
CA125 (U/ml)	0–35	27.24 ± 7.50	18.87 ± 7.79	4.77	<0.001
RCCA (mm)	≤1	1.02 ± 0.15	0.85 ± 0.11	5.48	<0.001
LCCA (mm)	≤1	0.96 ± 0.15	0.88 ± 0.12	2.34	0.02
DUO (ml)	–	400.00 (107.50, 1020.00)	525.00 (100.00, 1012.50)	−0.36	0.72
DPU (ml)	–	874.74 ± 161.22	837.63 ± 178.26	0.95	0.34
PET	–	0.58 ± 0.13	0.62 ± 0.11	−1.43	0.16
Kt/V r	–	0.42 (0.29, 0.49)	0.39 (0.28, 0.49)	−0.72	0.48
Kt/V p	–	1.39 ± 0.11	1.36 ± 0.14	1.11	0.27
Kt/V t	–	1.76 ± 0.21	1.71 ± 0.24	0.83	0.41
Ccr r	–	6.90 (3.65,12.24)	8.55 (3.67,13.05)	−0.63	0.53
Ccr p	–	43.00 ± 5.78	41.70 ± 6.88	0.90	0.37
Ccr t	–	50.60 ± 7.25	50.52 ± 8.95	0.04	0.97

In the 65 PD patients who received BIA, 35.4% (23/65) of the patients were overhydrated. The low sTβ4 group (32/65) showed a significantly lower level of PhA (5.42 ± 0.46 vs. 5.88 ± 0.41) (t=–4.28, *p* < 0.001) and a higher level of OH (2.60 [2.33, 2.78] vs. 2.20 [2.05, 2.40]) (Z=–3.24, *p* = 0.001) compared to the high sTβ4 group. Furthermore, OH was positively associated with hs-cTn (*r* = 0.281, *p* = 0.023).

### Correlation of sTβ4 with peritoneal permeability status determined by PET

The level of sTβ4 did not significantly differ among the low, low average, high average and high groups (*p* = 0.32), and it did not significantly differ between the low and low average groups or between the high and high average groups (*p* = 0.95) (Table 2). Moreover, Spearman’s correlation analysis showed that sTβ4 was not significantly associated with PET (*p* = 0.79).

**Table 2.** sTβ4 level in relation to PET.

Variable	L (*n* = 14)	LA (*n* = 33)	HA (*n* = 24)	H (*n* = 5)	F	*P*	LA and L (*n* = 47)	HA and H (*n* = 29)	Z	*p* Value
sTβ4 (ng/mL)	847.39± 362.69	1065.16± 403.25	1035.15± 460.53	832.90± 513.17	1.19	0.32	929.81 (605.51, 1300.88)	1000.23(546.52, 1430.97)	−0.07	0.95

### Correlation between sTβ4 and MIA indicators

**Figure 1.**
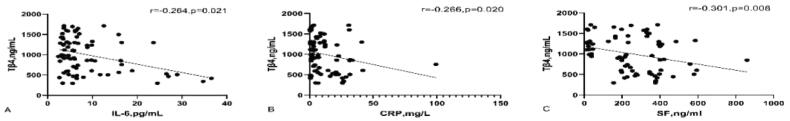
Correlations between sTβ4 and the **A** IL-6 (r=–0.264, *p* = 0.021), **B** CRP (r=–0.266, *p* = 0.020) and **C** SF (r=–0.301, *p* = 0.008) proinflammatory factors. Spearman’s correlation analysis was applied.

**Figure 2.**
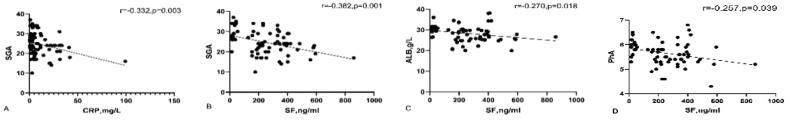
Correlations between pro-inflammatory factors and the following nutritional indices: **A** CRP and SGA (r=–0.332, *p* = 0.003); **B** SF and SGA (r=–0.382, *p* = 0.001); **C** SF and ALB (r=–0.270, *p* = 0.018); and **D** SF and PhA (r=–0.257, *p* = 0.039). Pearson’s or Spearman’s correlation analysis was applied.

**Figure 3.**
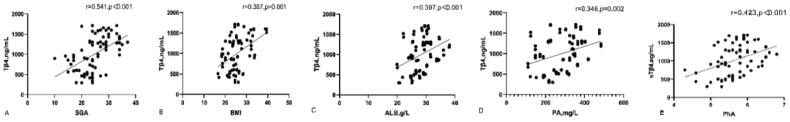
Correlations between sTβ4 and the following nutritional indices: **A** SGA (*r* = 0.541, *p* < 0.001); **B** BMI (*r* = 0.387, *p* = 0.001); **C** ALB (*r* = 0.397, *p* < 0.001); **D** PA (*r* = 0.346, *p* = 0.002); and **E** PhA (*r* = 0.423, *p* < 0.001). Spearman’s correlation analysis was applied.

Pearson’s or Spearman’s correlation analysis was performed to analyze the relationship between sTβ4 and MIA syndrome. Reduced sTβ4 was associated with high levels of the proinflammatory mediators, IL-6 (*p* = 0.021), CRP (*p* = 0.020) and SF (*p* = 0.008) (Figure 1). The level of the CRP proinflammatory mediator was inversely associated with SGA (*p* = 0.003), and the level of the SF proinflammatory mediator was inversely associated with SGA (*p* = 0.001), serum ALB (*p* = 0.018) and PhA (*p* = 0.039) (Figure 2). Furthermore, the sTβ4 level in PD patients was positively correlated with SGA (*p* < 0.001), BMI (*p* = 0.001), PhA (*p* < 0.001), serum ALB (*p* < 0.001) and serum PA (*p* = 0.002) (Figure 3). Moreover, sTβ4 was positively correlated with indicators involved in the evaluation of SGA (Table 3).

**Table 3.** Correlations between sTβ4 and SGA subgrades.

Variable	r	*p* Value
Weight variation	0.457	<0.001
Dietetic status	0.549	<0.001
Gastrointestinal symptoms	0.481	<0.001
Activity of daily living	0.363	0.001
Nutritional requirements	0.448	<0.001
Subcutaneous fat	0.333	0.003
Muscle wasting	0.403	<0.001

**Figure 4.**
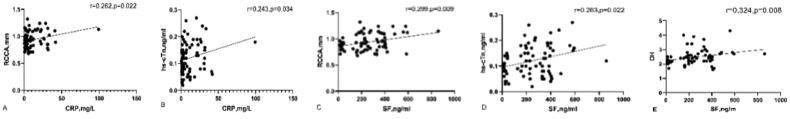
Correlations between proinflammatory factors and the following detrimental factors of AS and AS-related heart diseases: **A** CRP and RCCA (*r* = 0.262, *p* = 0.022); **B** CRP and hs-cTN (*r* = 0.243, *p* = 0.034); **C** SF and RCCA (*r* = 0.299, *p* = 0.009); **D** SF and hs-cTN (*r* = 0.263, *p* = 0.022); and **E** SF and OH (*r* = 0.324, *p* = 0.008). Pearson’s or Spearman’s correlation analysis was applied.

**Figure 5.**
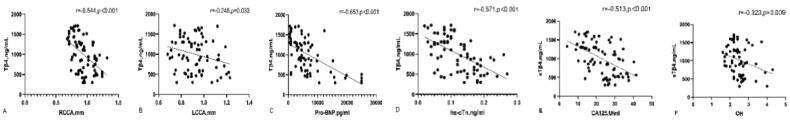
Correlations between sTβ4 and the following detrimental factors of AS and AS-related heart diseases: **A** RCCA (r=–0.544, *p* < 0.001); **B** LCCA (r=–0.246, *p* = 0.033); **C** Pro-BNP (r=–0.653, *p* < 0.001); **D** hs-cTN (r=–0.571, *p* < 0.001); **E** CA125 (r=–0.513, *p* < 0.001); and **F** OH (r=–0.323, *p* = 0.009). Spearman’s correlation analysis was applied.

**Figure 6.**
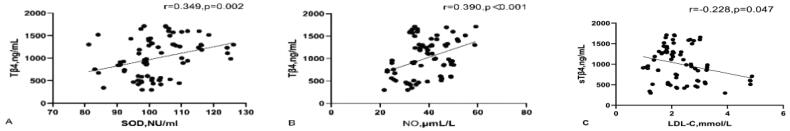
Correlations between sTβ4 and the following indicators involved in AS and AS-related heart disease: **A** SOD (*r* = 0.349, *p* = 0.002); **B** NO (*r* = 0.390, *p* < 0.001); and **C** LDL-C (r=–0.228, *p* = 0.047). Spearman’s correlation analysis was applied.

Regarding the factors involved in AS and AS-related heart diseases, the level of the CRP proinflammatory mediator was positively associated with those of RCCA intimal thickness (*p* = 0.022) and hs-cTN (*p* = 0.034); in addition, the level of SF was positively associated with the RCCA intimal thickness (*p* = 0.009) and hs-cTN level (*p* = 0.022) (Figure 4). Interestingly, the level of sTβ4 was inversely associated with those of detrimental factors, including RCCA intimal thickness (*p* < 0.001) and LCCA intimal thickness (*p* = 0.033), but positively associated with those of protective factors, such as SOD (*p* = 0.002) and NO (*p* < 0.001) (Figures 5 and 6).

### Association between sTβ4 and MIA incidence

The incidences of malnutrition (*p* < 0.001), inflammation (*p* = 0.029), atherosclerosis (*p* < 0.001), CVD (*p* < 0.001) and MIA syndrome (*p* = 0.026) were all significantly lower in the high sTβ4 group (Tables 1 and 4). Compared to PD patients without a history of CVD, PD patients with a history of CVD (33/76) had lower levels of sTβ4 (848.49 [507.39, 951.92] vs. 1279.21 [842.13, 1515.55] ng/mL) (*p* < 0.001).

**Table 4.** Incidences of malnutrition, inflammation, atherosclerosis and MIA syndrome in relation to sTβ4.

Variable	Low sTβ4 group (*n* = 38)	High sTβ4 group (*n* = 38)	χ^2^	*p* Value
Malnutrition	34/38(89.5%)	8/38(21.2%)	35.98	<0.001
Inflammation	18/38(47.4%)	8/38(21.2%)	5.85	0.029
Atherosclerosis	26/38(68.4%)	7/38(18.4%)	19.34	<0.001
MIA syndrome	13/38(34.2%)	4/38(10.5%)	6.14	0.026

### Association between sTβ4 levels in PD patients with and without CVD or MIA

The level of sTβ4 was significantly decreased in MIA PD patients compared to MIA-free PD patients (803.61 ± 335.94 vs. 1111.30 [605.51, 1043.64] ng/mL) (*p* = 0.034).

Univariate and multiple logistic regression analyses were performed to evaluate the influence of sTβ4 on MIA syndrome in PD patients in two models. In Model a, which analyzed the effect of sTβ4 in patients without MIA syndrome and those with all indicators of MIA syndrome, both univariate (OR = 0.997, 95% CI 0.994–0.999, *p* = 0.001) and multiple (OR = 0.996, 95% CI 0.993–0.999, *p* = 0.003) logistic regression analyses showed that the prevalence of MIA syndrome was significantly decreased in PD patients with increased levels of sTβ4. Moreover, in Model b, which analyzed the effect of sTβ4 in patients without MIA syndrome and those with at least one indicator of MIA syndrome, both univariate (OR = 0.997, 95% CI 0.995–0.998, *p* < 0.001) and multiple (OR = 0.997, 95% CI 0.995–0.998, *p* < 0.001) logistic regression analyses also showed that the prevalence of MIA syndrome was significantly decreased in PD patients with increased levels of sTβ4 (Table 5).

**Table 5.** Multiple logistic regression of the association between sTβ4 and MIA syndrome.

Model	MIA syndrome		
	sTβ4		
	OR	95% CI	*p* value
Model a	0.996	0.993–0.999	0.003
Model b	0.997	0.995–0.998	<0.001

## Discussion

The adverse effect of MIA syndrome on the prognosis of PD patients has been well described in previous studies [5,6]. Tβ4 has been demonstrated to be a protective mediator in various physiological processes, such as anti-inflammation, anti-fibrosis, cardio-protection and remodeling [8–12]. However, reports on the role of Tβ4 in ESKD patients, especially in PD patients, are limited. In addition, the tight association of sTβ4 with inflammation, nutritional status and AS in PD patients has never been previously explored. To our knowledge, this is the first study to demonstrate that a high Tβ4 level is associated with a low incidence of MIA syndrome in PD patients.

The present study achieved four encouraging findings. First, the sTβ4 level was inversely associated with the severity of inflammation in PD patients, implying that Tβ4 may also serve as an anti-inflammatory mediator in PD patients. Second, the sTβ4 level was directly and positively associated with nutritional status in PD patients. Third, sTβ4 represses the progression of AS and AS-related heart diseases. Fourth, sTβ4 may protect PD patients against MIA syndrome.

As a conserved plasmosin with a medium molecular weight of 4963.55 D, the transmembrane transport of Tβ4 is inhibited compared to that of substances with a low molecular weight, such as creatinine and urea nitrogen. Moreover, peritoneal fibrosis, which may significantly retard the transmembrane transport of medium molecular substances, is prevalent in PD patients, especially in those receiving stable PD treatment [15–17]. Thus, the transmembrane transport of Tβ4 from serum into dialysate in CAPD patients may be significantly reduced. To our knowledge, no studies have reported a significant association between peritoneal permeability and the level of sTβ4 in PD patients. Accordingly, the level of sTβ4 may not be significantly affected by peritoneal permeability in PD patients because only a small portion of sTβ4 is transported into the abdominal cavity *via* the peritoneum, which may account for the present finding that sTβ4 was not significantly associated with PET. Moreover, our other current studies found that dialysate Tβ4 mainly originates from peritoneal innate cells, preliminarily suggesting that the peritoneal permeability status is closely related to dialysate Tβ4, rather than sTβ4, in PD patients, but further exploration is required.

Inflammation occurs in approximately 30–50% of ESKD patients receiving PD, and it is usually triggered by uremic toxins, implanted exogenous catheters, glucose-based solutions, blood lipid metabolism disorders, oxidative stress, infection and immune response [18]. Consistently, 26/76 (34%) of the ESRD patients presented inflammation in the present study. Tβ4 has been demonstrated to be anti-inflammatory, suggesting that the sTβ4 level may be negatively correlated with the degree of inflammation in vital organs [10,19–21]. Tβ4 may counter inflammation through the following pathways. First, chemokines, such as meprin-α and POP, catalyze the cleavage of Tβ4 into AcSDKP. Second, Tβ4 may bind to the Tβ4-specific-binding receptor, Ku80, to fulfill its anti-inflammatory role [7]. Acting as a rate-limiting enzyme in the release of AcSDKP from Tβ4 [19,22,23], POP is highly activated in the case of inflammation, which in turn facilitates the cleavage of Tβ4 [19, 24–27]. In addition, the binding rate of Tβ4 with Ku80 also increases under inflammation [9]. These results indicate that Tβ4 metabolism may be enhanced under inflammatory conditions. In the present study, we found a strong inverse relationship between sTβ4 and the CRP, IL-6 and SF proinflammatory mediators. Fogo et al. showed that Tβ4 metabolism is enhanced as the production of proinflammatory mediators increases [24–27], which agreed with the present findings.

As an essential part of MIA syndrome, malnutrition is common in PD patients. Stevinkel et al. reported that approximately 50% of PD patients are classified as having malnutrition [5]. Accordingly, 55% (42/76) of patients were malnourished in the present study. As a core process in the progression of MIA syndrome, inflammation has a close association with the progression of malnutrition. Inflammation suppresses dietary intake and stimulates energy consumption, thereby leading to malnutrition [28]. Accordingly, we observed a significantly inverse association between CRP and SGA as well as between SF and SGA, ALB or PhA. These findings agreed with previous reports that have demonstrated the causality between inflammation and malnutrition in PD patients [29]. The sTβ4 level, due to its positive association with SGA, BMI, serum ALB, PA and especially PhA, has been recognized as a novel indicator of nutritional state in PD patients [30,31]. Thus, Tβ4 indicates the levels of dietary intake and energy consumption, two factors that influenced the nutritional states of the present patients. Taken together, Tβ4 may relieve inflammation to increase dietary intake and energy consumption, thereby improving nutrition status in PD patients. Moreover, Savino et al. Dardenne et al. and El-Zayat et al. demonstrated that Tβ4 may interfere with nutritional status *via* other pathways in addition to anti-inflammation [32–34]. Accordingly, in some of the present PD patients, malnutrition was related to sTβ4 but not to inflammatory factors. Thus, further investigation is required.

Drepper et al. reported that approximately 30% of PD patients are overhydrated [35]. Accordingly, 35.4% (23/65) of the patients were overhydrated in the present study. The interaction between overhydration and inflammation is complicated in PD patients. First, inflammation may be activated to induce injury to cardiac endothelial cells and enhance the calcification of vascular cells, leading to myocardial stunning, including myocardial ischemia and reperfusion, both of which may result in further complications, such as CVD, cardiac dysfunction and overhydration [6,36]. Accordingly, we showed a significant association of SF with OH as well as a significant association of hs-cTn with CRP and SF. Moreover, inflammation may contribute to malnutrition, which may reduce plasma colloid osmotic pressure but increase capillary permeability, both of which may contribute to overhydration. Additionally, overhydration in PD patients may contribute to uremic myocardiopathy, including heart failure and myocardial damage, as manifested by the stenosis or even occlusion of coronary arteries and injury of cardiac cells that may lead to myocardial stunning; subsequently, inflammation occurs with the activation of inflammatory cells, such as neutrophils and lymphocytes, as well as the overexpression of inflammatory factors, including CRP and IL-6[37,38].

Inflammation induces abnormal endothelium-dependent vascular relaxation, oxidative stress and lipid metabolism disorders, all contributing to the intimal thickness of the right and left carotid arteries as well as the emergence of AS-related heart diseases, including CVD [39,40]. In parallel with this mechanism, PD patients in the low sTβ4 group were at an increased risk of developing CVD. Additionally, compared to those without a history of CVD, PD patients with a history of CVD had a lower level of sTβ4. We also found that the levels of the CRP and SF proinflammatory factors were positively associated with those of the AS-related factors, RCCA intimal thickness and hs-cTN. Moreover, SF was also positively associated with OH, a novel hyperhydration index [30,31]. Smart et al. observed that a high Tβ4 level inhibits the progression of ischemic heart diseases [41–44]. Accordingly, we detected that the level of Tβ4 was inversely associated with the levels of indicators of AS and AS-related heart diseases in PD patients, such as RCCA intimal thickness, LCCA intimal thickness, pro-BNP level and hs-cTN level. Moreover, the levels of OH and serum CA125, markers of overload in peritoneal dialysis patients [30,31,45], were inversely associated with sTβ4, providing additional evidence that Tβ4 may exert a protective role in AS-related heart failure. Thus, we hypothesized that Tβ4 suppresses inflammation to impede the development of AS and AS-related heart diseases.

To drive the progression of AS and AS-related heart diseases, inflammation induces abnormal endothelium-dependent vascular relaxation, oxidative stress and lipid metabolism disorders [39–43]. Tβ4 has been shown to rectify these processes *via* its anti-inflammatory effects [41–43,46,47]. As expected, there was a strong correlation between sTβ4 and the AS-related factors, NO, SOD and LDL-C. Thus, Tβ4 may correct abnormal endothelium-dependent vascular relaxation, oxidative stress and lipid metabolism disorders to impede the progression of AS and AS-related heart diseases. Additionally, as inflammation may result in the fibrosis of myocardial cells and the loss of cardiac function, the inhibitory effects against fibrosis, oxidative stress and inflammation of Tβ4 may benefit the cardiac function protection [42,43,48–50].

The incidences of malnutrition, inflammation, AS, CVD and MIA syndrome were significantly decreased in patients with high sTβ4 levels. Moreover, the level of sTβ4 significantly decreased in MIA PD patients compared to MIA-free PD patients. Furthermore, both univariate and multivariate logistic regression analyses showed that the prevalence of MIA syndrome was significantly decreased in PD patients with increased levels of sTβ4 in the two models in the present study. Considering its inhibitory effects on malnutrition, inflammation, AS, CVD and MIA syndrome, the present findings may suggest that Tβ4 is a protective factor for PD patients.

## Limitations

First, the present study was a single-center study, which limited the significance of the present findings. Second, the levels of cytokines were measured only once at enrollment due to the cross-sectional design. Further well-designed cohort studies are needed to define the association of sTβ4 with MIA syndrome and the prognosis of PD patients based on data measured over the long term.

## Conclusions

A close interaction exists between sTβ4 and MIA syndrome in PD patients. The sTβ4 level decreases in PD patients with MIA syndrome. Moreover, the prevalence of MIA syndrome decreases significantly as the level of sTβ4 increases in PD patients. Tβ4 may serve as a protective factor for PD patients with MIA syndrome. The present findings may provide a new direction for further research into this area.

## Ethical approval

The present study was performed in accordance with the Declaration of Helsinki. The study protocol was approved by the Ethics Committee of Lianyungang Clinical College of Nanjing Medical University/The First People’s Hospital of Lianyungang (Registration No. LW-20220708001). Written informed consent was obtained from all the subjects participating in the study.

## Supplementary Material

Supplemental Material

## Abbreviations

PD: peritoneal dialysis
ESKD: end-stage kidney disease
AS: atherosclerosis
CVD: cardiovascular disease
MIA: malnutrition-inflammation-atherosclerosis
Tβ4: thymosin β4
CAPD: continuous ambulatory peritoneal dialysis
SH: smoking history
SBP: systolic blood pressure
DBP: diastolic blood pressure
SGA: subjective global nutritional assessment
BMI: body mass index
HGB: hemoglobin
Ret: reticulocyte
CRP: C reaction protein
IL-6: interleukin-6
GLU: glucose
Scr: serum creatinine
BUN: urea nitrogen
ALB: albumin
PA: prealbumin
Ca: calcium
P: phosphorus
iPTH: intact parathyroid hormone
CH: total cholesterol
TG: triglyceride
HDL-C: high-density lipoprotein cholesterol
LDL-C: low-density lipoprotein cholesterol
UA: uric acid
AB: actual bicarbonate
TIBC: total iron binding capacity
TRF: transferrin
SF: serum ferritin
SI: serum iron
β2-M: β2-microglobulin
FF: free fatty acid
HCY: homocysteine
SOD: superoxide dismutase
NO: Nitric Oxide
NGAL: neutrophil gelatinase-associated lipocalin
BNP: brain natriuretic peptide
pro-BNP: pro-brain natriuretic peptide
hs-cTN: high sensitive troponin
RCCA: right common carotid artery
LCCA: left common carotid artery
DUO: daily urine output
DPU: daily peritoneal ultrafiltration
PET: peritoneal equilibration test
Kt/V p: peritoneal weekly Kt/V
Kt/V r: residual kidney weekly
Kt/V t: total weekly Kt/V
Ccr p: peritoneal Ccr
Ccr r: kidney Ccr
Ccr t: total Ccr
BIA: bioimpedance analysis
PhA: phase angle
OH: overhydration index.
