# Supplementary material for: Association of serum thymosin β4 with malnutrition-inflammation-atherosclerosis syndrome in peritoneal dialysis patients: a cross-sectional study
Source: Ren Fail. 2023 May 3;45(1):2202761. doi: 10.1080/0886022X.2023.2202761 (PMC10158543; doi:10.1080/0886022X.2023.2202761)
Supplement: Supplemental Material [file IRNF_A_2202761_SM0759.zip › Editing_Certificate.pdf]

To whom it may concern,

We hereby confirm that the article **"Association of Serum Thymosin β4 with Malnutrition-Inflammation-AtherosclerosisSyndrome in Peritoneal Dialysis Patients: A Cross-Sectional Study",** written by Jiakun Tian, *et al.*, has been grammatically revised by **Shijiazhuang Longwin Translation Service Co., Ltd.** **(http://www.longwin58.com)**. For any linguistic questions regarding this article, please do not hesitate to contact us.

Best regards,

Shijiazhuang Longwin Translation Service Co.

29/December/2022
