# Supplementary material for: Association of serum thymosin β4 with malnutrition-inflammation-atherosclerosis syndrome in peritoneal dialysis patients: a cross-sectional study
Source: Ren Fail. 2023 May 3;45(1):2202761. doi: 10.1080/0886022X.2023.2202761 (PMC10158543; doi:10.1080/0886022X.2023.2202761)
Supplement: Supplemental Material [file Response_Letter.pdf › Response_Letter.pdf]

Dear Professor Fülöp,

Thank you for your email on December 5, 2022 and for the reviewers' comments concerning our manuscript entitled "Association of Serum Tβ4 with Malnutrition-Inflammation-Atherosclerosis Syndrome in Peritoneal Dialysis Patients: A Cross-Sectional Study" (ID:223049008). Those comments are all valuable and very helpful for revising and improving our paper. We have studied comments carefully and have made correction which we hope meet with approval. Revised portion are marked in yellow in the paper. The main corrections in the paper and the responds to the reviewer's comments are as flowing:

Responds to the reviewer's comments:

Response to reviewer #1:

1. Comment 1: The introduction section is short. It would be helpful to provide more information about Tβ4.

Response: Thanks for your comment. We have made corrections according to the Reviewer's comments. More information about Tβ4 concerning cellular functions, animal models, and clinical trials were provided in the Introduction section.

2. Comment 2: The relationship between the peritoneal permeability status determined by PET test and Tβ4 should be given. It would be useful to classify the patients according to their peritoneal membrane permeability status and compare the Tβ4 levels of the groups. Because the passage of Tβ4 into the peritoneal fluid will affect its serum level. Peritoneal clearance of Tβ4 should be discussed. In addition, PD duration of patients is one of the most important parameters determining inflammation. The fact that the PD times are not different is a contrast to this situation. How do the authors explain the lack of association of Tβ4 with PD duration? In addition, it should be stated whether the patients use icodextrin.

Response: Thanks for your comment. (1) As suggested, we analyzed the correlation between sTβ4 and peritoneal permeability status determined by PET and discussed the transportation of Tβ4 by peritoneum in ESRD patients receiving CAPD. Also, some novel findings were added.

(2) As suggested, PD duration is one important parameter related to inflammation, and Tβ4 is closely implicated in anti-inflammation. Moreover, Tβ4 is also involved in anti-fibrosis, cardio-protection and heart repair, all of which have been proved by other studies. Indeed, our study showed sTβ4 is associated with nutritional status, atherosclerosis, as well as CVD in PD patients. So, there may be many confounding factors interfering the relationship between sTβ4 and PD duration, despite that PD duration is an important mediator. Also, our single-center, cross-sectional study may be another reason for the lack of association. We suppose a further prospective cohort, multi-center study may provide a more persuasive result.

(3) The information of icodextrin-based PD solution was added.

3. Comment 3: How the subcutaneous fat measurements of the patients were made should be stated in the method section.

Response: Thanks for your comment. We have made correction according to the Reviewer's comments.

4. Comment 4: Kt/Vp: Is Plasma Kt/V? or is it peritoneal Kt/V? Why is total Kt/V not equal to the sum of peritoneal and renal Kt/V? How do the authors explain the low renal Kt/V in the group with higher daily urine output? Re-evaluation of these data is recommended.

Response: Thanks for your comment. (1) Given that Kt/Vp is peritoneal Kt/V, we have made the correction according to the comment.

(2) Considering the Reviewer's suggestion, we have re-evaluated our data. We are very sorry for our incorrect calculation of total Kt/V based on the plus of peritoneal and renal Kt/V, and we have made correction according to the Reviewer's comments.

(3) We re-evaluated our data and found no error in the data of DUO and Kt/V r in our study. Indeed, in the whole patients, Kt/V r was closely positively associated with DUO. When patients were divided into two groups according to DUO, those in the group of lower levels of DUO had significant decreased levels of Kt/V r ($Z=-6.25$, $P<0.05$). Also Spearman's correlation analysis proved that Kt/V t was positively associated with DUO ($r=0.90$, $P<0.001$).

However, when our patients were divided into two groups according to the level of sTβ4. Low renal Kt/V in the group was accompanied by higher daily urine output. The reasons are as follows: First, as the P values were 0.72 and 0.48, respectively, the differences in DUO and Kt/V r between the lower and higher sTβ4 groups in our study were not significant. Second, the range of DUO varied greatly among PD patients, which may result in variances. Also, the limited numbers of patients enrolled in our single-center, cross-sectional study may lead to variances. We suppose that a further well-designed cohort study may provide a more convincing result.

5. Comment 5: The article has many grammatical and spelling errors. Authors are required to review the article again in terms of spelling and spelling rules.

Response: Thanks for your comment. The revised manuscript has be edited through a competent English Editing Company.

6. Comment 6: There is a deficiency in the numerical expressions given in parentheses in the last paragraph of the Results section and the P value should be given as the true value.

Response: Thanks for your comment. We have made corrections according to the Reviewer's comments.

7. Comment 7: There are a lot of spelling mistakes in the discussion section.

Response: Thanks for your comment. The revised manuscript has been edited through a competent English Editing Company.

8. Comment 8: References need to be rearranged according to journal rules. (references 2,3,14,15, etc.). References 9 and 17 are the same.

Response: Thanks for your comment. We have made corrections according to the Reviewer's comments.

Response to reviewer #2:

1. Comment 1: In the Methods section, the authors have only included the list of routine markers analyzed (p. 2 L. 39-53). This is not sufficient. First, many of the listed indicators are not biochemical, and second, the methods of investigation for each of them should be given (they can be grouped);

Response: Thanks for your comment. We have re-written this part according to the Reviewer's suggestion.

2. Comment 2: The abbreviations for median and interquartile ranges should be added (p.3. L 11).

Response: Thanks for your comment. We have made corrections according to the Reviewer's suggestion.

3. Comment 3: Please arrange the images in Figure 1 in alphabetical order.

Response: Thanks for your comment. We have made corrections according to the Reviewer's comments.

Response to reviewer #3:

1. Comment 1: I recommend improving the format of references 2,3,14 and 15.

Response: Thanks for your comment. We have made corrections according to the Reviewer's comments.

2. Comment 2: Between lines 27 and 30 they comment on the glucose solutions used. They say that they use Baxter's solutions but the concentrations of 1.5% and 2.5% are those used in Fresenius. On the other hand, if they are Baxter's solutions, I don't know if they use overnight stays with Extraneal solution.

Response: Thanks for your comment. Baxter's glucose solutions at concentrations of 1.5%, 2.5% and 4.25% have been used as the main solutions for PD in China, either for daily treatment or for the evaluation of PET and dialysis adequacy. Here, we provide the Medicine Specifications. Unfortunately, no patients received Extraneal solution because it is unavailable during the period of our study

3. Comment 3: In the variables analyzed, no cardiovascular history of the patients was analyzed.

Response: Thanks for your comment. The association between the history of CVD (cardiovascular disease disease) and the level of Tβ4 was added.

4. Comment 4: It would also be interesting to have analyzed the CA-125 biomarker. Its role as a marker of overload is especially interesting in peritoneal dialysis patients.

Response: Thanks for your comment. Considering the Reviewer's suggestion, we have analyzed the CA-125 biomarker in the Methods, Results and Discussion sections.

5. Comment 5: In the results section, KtV of urea is not too high in both groups. It would have been interesting to add weekly creatintine clearance as another marker of adequate dialysis dose.

Response: Thanks for your comment. As Reviewer suggested, KtV of urea is not too high in both groups in the present study. The KtV t should be no less than 1.7 according to the Pertoneal Dialysis Standard Operating Procedure of China. Thus, weekly creatintine clearance as another marker of adequate dialysis dose. We have made corrections.

6. Comment 6: In the results section, it would have been interesting to analyze the role of sTβ4 in patients with high and low cardiovascular risk.

Response: Thanks for your comment. Considering the Reviewer's suggestion, we have analyzed the role of sTβ4 in patients with and without history of CVD.

7. Comment 7: In general, the discussion is correct. It would have been interesting to have completed the discussion with the role of bioimpedance. The role it contributes when addressing nutritional status and as a complementary study is well known. I attached a bibliographical reference: Mushnick R, Fein P, Mittman N, Goel N, Chattopadhyay J, Avram M. Relationship of bioelectrical impedance parameters to nutrition and survival in peritoneal dialysis patients. Kidney International. 2003;64:S53-S56.

Response: Thanks for your comment. Considering the Reviewer's suggestion, we learned these references, and selected 65 PD patients to receive the Multifrequency Bioimpedance Assessment (BIA). The nutritional status indicators Phase angle (PhA) and overhydration index (OH) were analyzed in our study and their significances were discussed.

Special thanks to you for your professional comments.

Response to reviewer #4:

1. Comment 1: English needs editing.

Response: Thanks for your comment. The revised manuscript has been edited by a competent English Editing Company.

2. Comment 2: More elaboration is needed to emphasize the known clinical use of Thymosin β4.

Response: Thanks for your comment. Considering the Reviewer's suggestion, more elaboration bout the known clinical use of thymosin β4 was added.

3. Comment 3: Medications esp those with anti-inflammatory effect significantly affect Thymosin β4 levels. Reports that NSAIDs induce thymosin beta-4 expression in a time- and concentration-dependent manner. Were any of the patients on NSAIDs? This is a major concern.

Response: Thanks for your comment. Considering the reviewer's suggestion, we counted our PD patients using NSAIDs. However, only a few were on NSAIDs. Our ESRD patients receiving PD treatment were at higher risks of developing gastrointestinal complications, especially gastrointestinal erosions, hemorrhage and even perforation because of accumulation of uremic toxins and overhydration. Unfortunately, one main side effect of NSAIDs is gastrointestinal complications, especially hemorrhage. Thus, NSAIDs may lead to more severe gastrointestinal complications and even death in PD patients. That may be the main reason for the result. Indeed, most of our PD patients took moderate doses of clopidogrel or apixaban when needed.

4. Comment 4: Peritoneal permeability of Thymosin β4 in patients is unclear.

Response: Thanks for your comment. As Reviewer suggested, peritoneal permeability of Thymosin β4 was added in the results and discussion sections.

5. Comment 5: Correlation of PD duration, cardiovascular morbidities with Thymosin β4 level and concentration is unclear. There are existing reports of Thymosin β4 promoting cardiomyocyte migration, survival, and repair.

Response: Thanks for your comment. (1) Correlation of PD duration and Thymosin β4 level in our study was positive, but not significant. The main reasons may be the following: First, PD duration of patients is one important parameter related to inflammation and Tβ4 is closely implicated in anti-inflammation. Moreover, Tβ4 is also involved in anti-fibrosis and cardio-protection and heart repair, all of which have been proved by other studies. Also, our study showed Tβ4 is associated with nutritional status and atherosclerosis as well. There may be many factors affecting the relationship between PD duration and the level of sTβ4. Thus, the level of sTβ4 in PD patients is affected by many factors, except for PD duration. Second, our single-center, cross-sectional study may be another reason affecting the association. We suppose a further prospective cohort study may provide a more convincing result.

(2) Considering the Reviewer's suggestion, we have added the correlation between history of CVD with Thymosin β4 level and concentration in the Results and Discussion Sections.

6. Comment 6:Did the calculated Kt/v include UOP (it should).

Response: As Reviewer suggested, the calculation of Kt/v t is based on the levels of UOP, so it was in our study. Indeed, in the whole patients, Kt/V r was closely positively associated with DUO. When our patients were divided into two groups according to DUO, those in the group of lower levels of DUO had significantly lower levels of Kt/V r ($Z=-6.25, P < 0.05$). Also Spearman's correlation analysis proved that Kt/V t was significantly associated with DUO ($r=0.90$, $P < 0.001$).

6. Comment 6: Vision of any future clinical implementation of Thymosin β4 in real practice should be added by the authors.

Response: Thanks for your comment. We have made corrections according to the Reviewer's suggestion.

7. Comment 7: Clarification regarding different types of solutions used is needed.

Response: Thanks for your comment. The Medicine Specifications of glucose-based PD solution of various concentrations (1.5%, 2.5% and 4.25%) (Baxter Inc.,) were provided. Information about icodextrin-based PD solution was also added.

Thank you very much for your professional comments and suggestions

We tried our best to improve the manuscript and made some changes in the manuscript.

These changes will not influence the content and framework of the paper. And here we marked the changes in yellow as requested.

We appreciate for Editors/Reviewers' warm work earnestly, and hope that the correction will meet with approval.

Thank you very much for your comments and suggestions.

Sincerely yours,

Jiakun Tian, MD
Department of Nephrology, Shanghai General Hospital of Nanjing Medical University, Shanghai, China
Shanghai, 200080
China
tianjiakun03@163.com
