# Supplementary material for: Association of serum thymosin β4 with malnutrition-inflammation-atherosclerosis syndrome in peritoneal dialysis patients: a cross-sectional study
Source: Ren Fail. 2023 May 3;45(1):2202761. doi: 10.1080/0886022X.2023.2202761 (PMC10158543; doi:10.1080/0886022X.2023.2202761)
Supplement: Supplemental Material [file Response_Letter_R2..pdf › Response_Letter_R2..pdf]

Dear Professor Fülöp,

Thank you for your email on January 25, 2023 and for the Editors/Reviewers' comments concerning our manuscript entitled "Association of Serum Tβ4 with Malnutrition-Inflammation-Atherosclerosis Syndrome in Peritoneal Dialysis Patients: A Cross-Sectional Study" (ID:223049008.R1). Those comments are all valuable and very helpful for revising and improving our paper. We have studied comments carefully and have made correction which we hope meet with approval. Revised portion are marked in yellow in the paper. The main corrections in the paper and the responds to the reviewer's comments are as flowing:

Responds to the Editors and reviewer's comments:

Response to Editors:

1. Comment 1: Current manufacturer of SPSS is not " ; SPSS Inc." but IBM. Please correct.

Response: Thanks for your comment. We have made the correction according to comments.

2. Comment 2: Please update terminology and nomenclature to be in keeping with most recent KDIGO consensus recommendation, including referring to "kidney" rather than "renal" in text (for example, "ESKD" rather than "ESRD"; "RRT-dependence" replace with "KRT-dependence"), please refer to paper: PMID 32619132. Ren Fail. 2020 Nov;42(1):560-566.

Response: Thanks for your comment. We have updated terminology and nomenclature to keep with most recent KDIGO consensus recommendation.

3. Comment 3: Write-up is needlessly bulky and shortened be shortened in Results section; Authors do not list all statistically significant values, especially from univariate correlations - mention only key point and otherwise refer readers to Tables.Are the Authors able to offer multiple adjusted model to confirm this novel association?

Response: Thanks for your comment. Univariate and multiple logistic regression analyses were performed to evaluate the influence of sTβ4 on MIAs in PD patients in Model a and Model b in the revised manuscript.

Response to reviewer #1:

1. Comment 1: The true value of the P value should be given (not as P<0.05 or P>0.05) in the entire article, including the abstract.

Response: Thanks for your comment. We have given the true value of the P value.

2. Comment 2: In order to create a common language in the article, only one of the expressions kidney or renal should be used.

Response: Thanks for your comment. We used the the expression of kidney in the revised manuscript.

Response to reviewer #2:

Comment : The authors have made significant changes that have greatly improved the quality of the manuscript. I have no further comments and recommend the manuscript for publication.

Response: Thanks for your comment. We appreciated your recommendation so much.

Response to reviewer #3:

Comment : First, I would like to congratulate the authors of the work. I think it is always important to assess new prognostic biomarkers in peritoneal dyalisis patients.The authors have added the modifications that I proposed in the previous comment. Good job.

Response: Thanks for your comment. We are very grateful of your kind approval of our job.

Thank you very much for your professional comments and suggestions

We have improved the manuscript and made some changes in the manuscript. And we marked the changes in yellow as requested.

We appreciate for Editors/Reviewers' warm work earnestly, and hope that the corrections will meet with approval.

Thank you very much for your comments and suggestions.

Sincerely yours,

Jiakun Tian, MD
Department of Nephrology, Shanghai General Hospital of Nanjing Medical University, Shanghai, China
Shanghai, 200080
China
tianjiakun03@163.com
