# Supplementary material for: Association of serum thymosin β4 with malnutrition-inflammation-atherosclerosis syndrome in peritoneal dialysis patients: a cross-sectional study
Source: Ren Fail. 2023 May 3;45(1):2202761. doi: 10.1080/0886022X.2023.2202761 (PMC10158543; doi:10.1080/0886022X.2023.2202761)
Supplement: Supplemental Material [file Response_Letter_R3.pdf › Response_Letter_R3.pdf]

Dear Professor Fülöp,

Thank you for your email on February 2, 2023 and for the editor' comments concerning our manuscript entitled "Association of Serum Tβ4 with Malnutrition-Inflammation-Atherosclerosis Syndrome in Peritoneal Dialysis Patients: A Cross-Sectional Study" (ID:223049008.R2). Those comments are very helpful for revising and improving our paper. We have studied comments carefully and have made corrections which we hope meet with approval. The main corrections in the paper and the responds to the editor's comments are as flowing:

1. Comment 1: Please place Table and Figures each on separate pages, to make processing easier after acceptance

Response: Thanks for your comment. We have made corrections according to the comments.

2. Comment 2: Please list Legend, Abbreviation etc., separately for figures, not part of the figure

Response: Thanks for your comment. We have listed Legend, Abbreviation etc., separately for figures.

3. Comment 3: May I ask for a further revision of Abstract, however. Describe the cohort main demographic, etc. characteristics, in Abstract In disagreement with Reviewer #1, this Editor does not believe all the univariate associations should be described in great detail in Abstract; would limit to key points only (may be 1/3rd - 1/4th of current length, with regard to describing univariate relationships… just emphasize a few key ones, with true p values) On the other hand, would you be able to show results and key OR with 95% CIs, and p values, from multiple adjusted models.

Response: Thanks for your comment. We have described relationships between Tβ4 and main demographic characteristics according to the editors's comments in Abstract. The univariate associations were limited to key characteristics only. Also, results and key OR with 95% CIs, and p values from multiple adjusted models were provided in Abstract.

Thank you very much for your professional comments and suggestions

We tried our best to improve the manuscript and made some changes in the manuscript. We appreciate for Editors/Reviewers' warm work earnestly, and hope that the corrections will meet with approval.

Thank you very much for your comments and suggestions.

Sincerely yours,

Jiakun Tian, MD
Department of Nephrology, Shanghai General Hospital of Nanjing Medical University, Shanghai, China
Shanghai, 200080
China
tianjiakun03@163.com
