# Supplementary material for: Association of serum thymosin β4 with malnutrition-inflammation-atherosclerosis syndrome in peritoneal dialysis patients: a cross-sectional study
Source: Ren Fail. 2023 May 3;45(1):2202761. doi: 10.1080/0886022X.2023.2202761 (PMC10158543; doi:10.1080/0886022X.2023.2202761)
Supplement: Supplemental Material [file Response_Letter_R4.pdf › Response_Letter_R4.pdf]

Dear Professor Fülöp,

Thank you for your email on Feb 20, 2023 and for the reviewers' comments concerning our manuscript entitled "Association of Serum Tβ4 with Malnutrition-Inflammation-Atherosclerosis Syndrome in Peritoneal Dialysis Patients: A Cross-Sectional Study" (ID:223049008). Those comments are all valuable and very helpful for revising and improving our paper. We have studied comments carefully and have made correction which we hope meet with approval. Revised portion are marked in yellow in the paper. The main corrections in the paper and the responds to the reviewer's comments are as flowing:

Responds to the reviewer's comments:

Response to reviewer #1:

1. I have read your paper with great interest. I have a few minor observations. First, this is a cross-sectional study. Even with a positive result on logistic regression, I would be more careful in suggesting that Tβ4 is protective against MIA. Also, it appears that as high as 42 of your 76 patients were malnourished while only 26 inflamed. I would understand that Tβ4 is anti-inflammatory, but why would it correlate with malnourished state without inflammation? Was there any correlation with adequacy measures?

Response: Thanks for your professional comment. Savino W, Dardenne M,and Salwa Refat El-Zayat have demonstrated that Tβ4 may influence the nutritional status via pathways other than anti-inflammation. And we discussed the relationship between Tβ and malnutrition unrelated to inflammation.

Also, our single-center, cross-sectional study may be another reason causing such an association. Another research is under way to investigate this association. We suppose our further prospective cohort, multi-center study may provide a more persuasive result.

2. Comment 2: Also, you fail to discuss the known association between inflammation and overhydration in PD patients and the triangle between overhydration, myocardial stunning and inflammation. What percentage of your patients were overhydrated?

Response: Thanks for your comment. We have made corrections according to the Reviewer's comments.

3. Comment 3: The language of your paper is largely clear, although some choice of words are not appropriate. Another review with a native English speaker may provide benefit.

Response: Thanks for your comment. We invited a specialist in English writing to improve the article.

4. Comment 4: Also, while introducing known effects of Tβ4 on fibrosis and organ function, you need to provide references for your statements. For instance, inhibitory effect relates to fibrosis and oxidative stress, but how does it relate to organ function?

Response: Thanks for your comment. We have made corrections according to the Reviewer's comments.

Thank you very much for your professional comments and suggestions

We tried our best to improve the manuscript and made some changes in the manuscript.

These changes will not influence the content and framework of the paper. And here we marked the changes in yellow as requested.

We appreciate for Editors/Reviewers' warm work earnestly, and hope that the correction will meet with approval.

Thank you very much for your comments and suggestions.

Sincerely yours,

Jiakun Tian, MD
Department of Nephrology, Shanghai General Hospital of Nanjing Medical University, Shanghai, China
Shanghai, 200080
China
tianjiakun03@163.com
