# Supplementary material for: Association of serum thymosin β4 with malnutrition-inflammation-atherosclerosis syndrome in peritoneal dialysis patients: a cross-sectional study
Source: Ren Fail. 2023 May 3;45(1):2202761. doi: 10.1080/0886022X.2023.2202761 (PMC10158543; doi:10.1080/0886022X.2023.2202761)
Supplement: Supplemental Material [file Response_Letter_R5.pdf › Response_Letter_R5.pdf]

Dear Professor Fülöp,

Thank you for your email on March 13, 2023 and for the Editors/Reviewers' comments concerning our manuscript entitled "Association of Serum Tβ4 with Malnutrition-Inflammation-Atherosclerosis Syndrome in Peritoneal Dialysis Patients: A Cross-Sectional Study" (ID:223049008.R4). Those comments are all valuable and very helpful for revising and improving our paper. We have studied comments carefully and have made corrections which we hope meet with approval. The main corrections in the paper and the responds to the reviewer's comments are as flowing:

Response to Editors:

1. Comment 1: The paper is well revised for scientific content

Response: Thanks for your professional comment so much. We are very grateful of your kind comment of our job.

2. Comment 2: The paper is not sufficiently revised for write--up; here are my comments:

"ABSTRACT. Results section still not written up in a sufficiently parsimonious manner; listing mpx p values, each of them separately… using up a lot of word counts. On the other hand, NONE of the cohort baseline main characteristics (age, sex, % diabetics, PD features) are mentioned any.

Part of the last sentence in Abstract (" …and Tβ4 may act as a protective mediator in PD patients. ") I cannot see it to be justified based of a cross-sectional study, examining mere correlations.

MAIN PAPER.

Paper should be described in a more parsimonious manner. Results section. Needlessly detailed – Authors do not need to list all significant p values; rather emphasize key point(s) and otherwise refer to Table 1. Same concerns regarding needly prolonged description of Table 3. Same concerns for prolonged description of Figure 5., 6.

Table 1. Avoid excessive abbreviation (UA, P, Alb, TC… etc.) – do impair eadability of the Tables (and many time do not save on word count). Please write these out preferably in full, unless truly log

List also normal values for the Authors' lab in the first Column.

Table 1 -2.; also noted missing spaces between numbers; numbers and letter (where they should be)

The entire Table should be carefully edited for neatness

Table 4 missing the Legends of abbreviation for this Table

All table should have proper Legends, if abbreviations used in Table"

Response: Thanks for your comment. We have made corrections according to the comments.

3. Comment 3: English professional Editing still needed; several incorrect expression (e.g.: "protopathy"), capitation errors still noted. These may seriously limit value of the paper if not done.

Response: Thanks for your comment. We invited the English Editing Company AJE to improve the article.

Thank you very much for your professional comments and suggestions. We tried our best to improve the manuscript and made some changes in the manuscript. These changes will not influence the content and framework of the paper.

We appreciate for Editors/Reviewers' warm work earnestly, and hope that the correction will meet with approval.

Thank you very much for your comments and suggestions.

Sincerely yours,

Jiakun Tian, MD

Department of Nephrology, Shanghai General Hospital of Nanjing Medical University, Shanghai, China
Shanghai, 200080
China
tianjiakun03@163.com
