# Supplementary material for: Association of serum thymosin β4 with malnutrition-inflammation-atherosclerosis syndrome in peritoneal dialysis patients: a cross-sectional study
Source: Ren Fail. 2023 May 3;45(1):2202761. doi: 10.1080/0886022X.2023.2202761 (PMC10158543; doi:10.1080/0886022X.2023.2202761)
Supplement: Supplemental Material [file Response_Letter_R6.pdf › Response_Letter_R6.pdf]

Dear Professor Fülöp,

Thank you for your email on March 29, 2023 and for the editors' comments concerning our manuscript entitled "Association of Serum Tβ4 with Malnutrition-Inflammation-Atherosclerosis Syndrome in Peritoneal Dialysis Patients: A Cross-Sectional Study" (ID:223049008). Those comments are all valuable and very helpful for revising and improving our paper. We have studied comments carefully and have made correction which we hope meet with approval. The main corrections in the paper and the responds to the reviewer's comments are as flowing:

Responds to the reviewer's comments:

Response to Editor:

1. Comment 1: Some concerns remain. As stated in our former letter: ABSTRACT: " …On the other hand, NONE of the cohort baseline main characteristics (age, sex, % diabetics, PD features) are mentioned any " Part of the last sentence in Abstract (" …and Tβ4 may act as a protective mediator in PD patients. ") I cannot see it to be justified based of a cross-sectional study, examining mere correlations.

Response: Thanks for your professional comments. Description of the cohort baseline main characteristics including sex, primary disease, age and PD features were added in the abstract. Modification of the the last sentence in Abstract was also made according to the comments.

2. Comment 2: Please remove all editing tracking marks and side "comments" (still do show up in Words).

Response: Thanks for your comment. We have made corrections according to the comment.

3. Comment 3: Some expression still not interpretable in contemporary English: "protopathic sensation" – please correct.

. Response: Thanks for your comment. We have made corrections according to the comment.

Thank you very much for your professional comments and suggestions.We tried our best to improve the manuscript and made some changes in the manuscript. These changes will not influence the content and framework of the paper. We appreciate for Editors/Reviewers' warm work earnestly, and hope that the corrections will meet with approval.

Sincerely yours,

Jiakun Tian, MD
Department of Nephrology, Shanghai General Hospital of Nanjing Medical University, Shanghai, China
Shanghai, 200080
China
tianjiakun03@163.com
